# Assessment of Immature Platelet Fraction in the Diagnosis of Wiskott–Aldrich Syndrome

**DOI:** 10.3389/fped.2015.00049

**Published:** 2015-06-01

**Authors:** Robert Sokolic, Neal Oden, Fabio Candotti

**Affiliations:** ^1^Office of Cellular, Tissue and Gene Therapies, Center for Biologics Evaluation and Research, Food and Drug Administration, Silver Spring, MD, USA; ^2^Emmes Corporation, Rockville, MD, USA; ^3^Disorders of Immunity Section, Genetics and Molecular Biology Branch, National Human Genome Research Institute, National Institutes of Health, Bethesda, MD, USA; ^4^Division of Immunology and Allergy, Centre Hospitalier Universitaire Vaudois, Lausanne, Switzerland

**Keywords:** thrombocytopenia, differential diagnosis, immature platelet fraction, Wiskott–Aldrich syndrome, immune thrombocytopenic purpura

## Abstract

Children with Wiskott–Aldrich syndrome (WAS) are often first diagnosed with immune thrombocytopenia (ITP), potentially leading to both inappropriate treatment and the delay of life-saving definitive therapy. WAS is traditionally differentiated from ITP based on the small size of WAS platelets. In practice, microthrombocytopenia is often not present or not appreciated in children with WAS. To develop an alternative method of differentiating WAS from ITP, we retrospectively reviewed all complete blood counts and measurements of immature platelet fraction (IPF) in 18 subjects with WAS and 38 subjects with a diagnosis of ITP treated at our hospital. Examination of peripheral blood smears revealed a wide range of platelet sizes in subjects with WAS. Mean platelet volume (MPV) was not reported in 26% of subjects, and subjects in whom MPV was not reported had lower platelet counts than did subjects in whom MPV was reported. Subjects with WAS had a lower IPF than would be expected for their level of thrombocytopenia, and the IPF in subjects with WAS was significantly lower than in subjects with a diagnosis of ITP. Using logistic regression, we developed and validated a rule based on platelet count and IPF that was more sensitive for the diagnosis of WAS than was the MPV, and was applicable regardless of the level of platelets or the availability of the MPV. Our observations demonstrate that MPV is often not available in severely thrombocytopenic subjects, which may hinder the diagnosis of WAS. In addition, subjects with WAS have a low IPF, which is consistent with the notion that a platelet production defect contributes to the thrombocytopenia of WAS. Knowledge of this detail of WAS pathophysiology allows to differentiate WAS from ITP with increased sensitivity, thereby allowing a physician to spare children with WAS from inappropriate treatment, and make definitive therapy available in a timely manner.

## Introduction

Immune thrombocytopenia (ITP) is the most common cause of newly discovered isolated thrombocytopenia in childhood (1), with an incidence of 1.9–6.4 in 100,000 children per year, and a prevalence of 9.3 in 100,000 boys (2). ITP is also a diagnosis of exclusion (3). Therefore, in order to diagnose the most common cause of newly recognized thrombocytopenia in children, all less common causes must first be ruled out. This leads to a situation wherein the need for parsimonious use of diagnostic resources conflicts with the imperative to make a diagnosis by ruling out other diagnoses, potentially leading to premature closure in the diagnosis of boys newly found to be thrombocytopenic. Premature closure, a known source of diagnostic error (4), can lead to two potential adverse consequences – treatment for a disease that is felt to be present but, in fact, is not, and delay of diagnosis and treatment of the disease that is actually present. Extensive resources have been invested by the American Society of Hematology in developing guidelines to diagnose ITP expeditiously, attempting to minimize both misdiagnosis of less common forms of thrombocytopenia and potentially costly, low-yield, and/or invasive investigations (3).

Another cause of newly recognized thrombocytopenia in boys, from which ITP must be differentiated, is Wiskott–Aldrich syndrome (WAS). WAS is an X-linked genetic disease, of widely varying clinical severity, which includes aspects of immunodeficiency, autoimmunity, and cancer predisposition in addition to microthrombocytopenia. The timely diagnosis of WAS, particularly its distinction from ITP, is important for a number of reasons. Immunosuppression is a first-line treatment for ITP (3). This treatment carries an increased risk in immunodeficient patients with WAS. Failure to diagnose WAS can lead to the delay of appropriate treatment. With respect to definitive treatment, hematopoietic cell transplant (HCT) for WAS has been felt to be most efficacious in younger children (5–7). Similarly, gene therapy for WAS is suspected to be most efficacious in very young children (8) as is the case for a number of other primary immunodeficiencies (9–11). Failure to diagnose WAS in a patient with newly recognized thrombocytopenia can lead to mistakes of omission and commission that may be fatal.

Classically, WAS is distinguished from other forms of newly recognized thrombocytopenia by virtue of small platelet size (1, 2, 12). This is traditionally recognized on review of a peripheral blood smear, a key part of the evaluation of suspected ITP (3). Alternatively, modern hematology analyzers report the mean platelet volume (MPV) numerically.

Nevertheless, it is not always the case that patients with WAS have small platelets. Cases of WAS have been described with normal (13, 14) or even increased (15) MPV. Furthermore, platelet size in one WAS patient was reported to vary over time with the dose of immunoglobulin replacement (16).

Another problematic aspect of using MPV in the differential diagnosis of newly recognized thrombocytopenia is that the parameter is often difficult to measure in moderate to severe thrombocytopenia (17), which is precisely the clinical situation in which the differential diagnosis is most important.

In our own cohort of patients with WAS, data on date of onset of symptoms and date of diagnosis are available for 21 patients with no family history of WAS. For these patients, the average diagnostic delay was 5 years (median 1.5 years, range 9 days to 27 years). Alternative laboratory investigations that could suggest the diagnosis of WAS and lead to molecular testing in an acceptable minority of cases of newly recognized thrombocytopenia would be useful.

The number of platelet parameters available from modern hematology analyzers is greater than it has been in the past (17, 18). One parameter that is now commonly reported is the immature platelet fraction (IPF) (19–21). Immature platelets, like reticulocytes, are recognized by the persistence of RNA leading to reticulation on analysis (19, 21–23). As with the reticulocyte index in anemia, the IPF is thought to increase in thrombocytopenic states due to peripheral destruction of platelets and to be inappropriately normal in thrombocytopenia with impaired platelet production (23).

Wiskott–Aldrich syndrome has classically been described as a disease of platelet destruction similar to ITP (24). Nevertheless, data in patients with WAS and in Wiskott–Aldrich syndrome protein (WASp) knockout mice suggest that there is a component of decreased platelet production in the thrombocytopenia of WAS (25, 26). On this basis, we hypothesized that the IPF might be a useful parameter to add to the platelet count and MPV in the clinically important differential diagnosis of WAS and ITP. As a preliminary step in exploring this hypothesis, we retrospectively analyzed hematologic data collected in the course of routine care of patients with either WAS or ITP at the Clinical Center of the National Institutes of Health (CC NIH) in Bethesda, MD, USA. By combining the measure of IPF with the platelet count, we derived a rule to suggest when it might be appropriate to consider a diagnosis of WAS. When evaluated for a subsequent cohort of subjects, this rule showed appropriate diagnostic characteristics to be used as an inexpensive screening test for WAS.

## Materials and Methods

The use of diagnoses and platelet, MPV, and IPF values of subjects with ITP and WAS was exempted from IRB review by the hospital’s Office of Human Subjects Research (certificates BTRIS_2013_615_SOKOLIC_R_NHGRI and BTRIS_2014_711_SOKOLIC_R_NHGRI). Consent to use these data was obtained from the principal investigators of the protocols upon which the specimens were drawn.

Immature platelet fraction became a clinically reportable parameter at the CC NIH in May of 2009. All measures of platelet counts, MPVs, and IPF reported at the hospital from May of 2009 through March 26, 2013 in subjects with a discharge diagnosis of either WAS or ITP were collected using the Biomedical Translational Research Information System (BTRIS). Data were collected stripped of personal identifiers. The blood for these assays had initially been collected in the course of routine or investigational evaluation on protocols approved by the various institutional review boards of the NIH intramural research program.

Unique subject identifiers created by BTRIS were attached to each record and used to associate diagnoses with laboratory values for each blood draw. The frequency of absent data for MPV was calculated, as were average platelet counts for blood draws including and not including MPV. Single blood draws from which platelet count, MPV, and IPF were all reported, were selected for further analysis. Two strategies were used to prevent subjects with more frequent measurements from influencing the analysis more than did subjects with few or single measurements. One strategy was to average multiple values from single subjects. The other was to use only the first-blood draw for each subject. We refer to the data obtained using the first strategy as the per-subject average data set for the derivation cohort. We refer to the data set obtained using the second strategy as the first-blood-draw data set for the derivation cohort.

Using data from the per-subject average data set for the derivation cohort, an ROC curve was calculated for the diagnosis of WAS based on MPV and a separate ROC curve for the diagnosis of WAS was calculated based on IPF and platelet count. Using these curves and an *à priori* assumption that a 10% probability of a patient having WAS would be a reasonable cut off for further consideration of the diagnosis, a prediction rule was developed for diagnosing WAS based on platelet and IPF values (the IPF/PLT rule). We contrasted this rule with the classic rule of considering WAS in any patient with an MPV less than the lower limit of normal (the MPV rule).

In order to determine the diagnostic characteristics of the two rules, a second set of data was obtained from BTRIS. All measures of platelet counts, MPVs, and IPF reported at the hospital from April 2, 2013 through March 18, 2014 in subjects with a discharge diagnosis of either WAS or ITP were collected. As for the derivation cohort, two data sets were created for the validation cohort, a per-subject average data set and a first-blood-draw data set.

After obtaining the two data sets for the validation cohort, the MPV rule and the IPF/PLT rule were each used separately to determine a diagnosis of WAS or ITP for each subject. These diagnoses were then compared to the recorded discharge diagnoses to determine sensitivity and specificity. Fisher’s exact test was used to determine if each rule could significantly separate subjects with WAS from subjects with a diagnosis of ITP. ROC curves based on the per-subject average values in the validation cohort were then calculated.

Descriptive statistics and diagnostic characteristics of the two rules were calculated using Microsoft Excel for Macintosh 2011 version 14.3.8 or Microsoft Excel 2010 for Windows. Student’s *t*-tests and Fisher’s exact tests were performed using calculators available at www.graphpad.com. Logistic regressions, ROC curve analysis, and the prediction rule were all calculated using SAS.

## Results

### Platelet parameters for subjects with WAS and ITP

All data for the entire derivation cohort are presented in Table S1 in Supplementary Material. There were 1,365 blood draws for which a platelet count was available. In both subjects with a diagnosis of ITP and subjects with WAS, as well as in both groups together, the platelet counts were lower in CBCs that did not report an MPV as compared with CBCs that did report an MPV (Table 1). This difference was significant for subjects with a diagnosis of ITP and for the whole cohort, but only trended toward significance for the smaller group of WAS subjects. These data confirm that MPV is less likely to be reported when the platelet count is lower.

**Table 1:** **Comparison of blood draws reporting or not reporting an MPV in subjects with a diagnosis of ITP, in subjects with WAS, and in all subjects**.

	Number of blood draws		% with no MPV	Average platelet counts		*p*
	MPV reported	No MPV reported		MPV reported	No MPV reported	
ITP	954	343	26	118,000	33,000	<0.0001
WAS	57	11	16	85,000	45,000	0.0961
Total	1,011	354	26	116,000	34,000	<0.0001

Complete data, including a reported platelet count, MPV, and IPF percentage, were available for 160 blood draws from 38 subjects with a diagnosis of ITP and 27 blood draws from 18 subjects with WAS. These data are presented in Table S2 in Supplementary Material. The per-subject average data set and the first-blood-draw data set for the derivation cohort are presented in Table 2.

**Table 2:** **Final data sets for the derivation cohort**.

Subject	Diagnosis	Number of blood draws	Average (SD) of all blood draws			First-blood draw		
			Platelets/mcL	MPV, fL	IPF, %	Platelets/mcL	MPV	IPF, %
1	Immune thrombocytopenic purpura	1	74,000	14.50	28.50			
2	Immune thrombocytopenic purpura	1	91,000	10.60	3.80			
3	Immune thrombocytopenic purpura	2	86,000(16,000)	14.50(0.28)	14.90(1.27)	75,000	14.70	14.00
4	Wiskott–Aldrich syndrome	1	30,000	8.80	3.90			
5	Immune thrombocytopenic purpura	1	419,000	9.60	1.50			
7	Wiskott–Aldrich syndrome	1	204,000	9.60	1.40			
8	Immune thrombocytopenic purpura	4	11,000(2,000)	9.125(1.08)	22.38(6.28)	9,000	8.40	13.10
10	Immune thrombocytopenic purpura	1	198,000	13.10	10.10			
11	Immune thrombocytopenic purpura	4	138,000(28,000)	10.3(0.41)	3.73(0.67)	120,000	10.40	3.80
14	Wiskott–Aldrich syndrome	1	51,000	8.30	1.50			
16	Immune thrombocytopenic purpura	1	39,000	13.40	12.90			
17	Immune thrombocytopenic purpura	5	112,000(14,000)	10.62(0.43)	3.54(1.45)	96,000	11.20	4.90
18	Wiskott–Aldrich syndrome	1	240,000	8.70	0.60			
20	Immune thrombocytopenic purpura	10	280,000(55,000)	11.78(0.27)	5.3(1.32)	340,000	12.00	3.80
21	Immune thrombocytopenic purpura	1	191,000	9.60	2.30			
22	Immune thrombocytopenic purpura	5	135,000(15,000)	11.12(0.41)	4.96(0.86)	160,000	11.20	3.70
23	Wiskott–Aldrich syndrome	3	111,000(29,000)	9.60(0.46)	1.47(1.00)	129,000	9.20	0.70
24	Immune thrombocytopenic purpura	2	174,000(16,000)	10.95(0.28)	5.15(1.27)	162,000	10.90	5.00
25	Wiskott–Aldrich syndrome	1	171,000	8.70	1.80			
26	Wiskott–Aldrich syndrome	1	120,000	9.20	0.80			
28	Wiskott–Aldrich syndrome	1	103,000	9.40	1.90			
29	Immune thrombocytopenic purpura	1	386,000	10.00	1.60			
31	Immune thrombocytopenic purpura	1	109,000	10.60	6.20			
32	Immune thrombocytopenic purpura	2	59,000(42,000)	10.80(0.80)	4.85(8.47)	29,000	11.60	7.10
33	Immune thrombocytopenic purpura	1	249,000	11.00	7.83			
34	Wiskott–Aldrich syndrome	1	60,000	7.50	0.70			
35	Immune thrombocytopenic purpura	1	72,000	10.20	5.90			
37	Immune thrombocytopenic purpura	1	23,000	12.80	7.10			
38	Wiskott–Aldrich syndrome	2	183,000(12,000)	9.55(0.42)	3.15(1.23)	191,000	10.10	2.90
39	Immune thrombocytopenic purpura	35	200,000(78,000)	10.29(0.42)	5.46(1.22)	329,000	9.50	4.40
40	Immune thrombocytopenic purpura	9	183,000(22,000)	10.44(0.20)	2.63(0.78)	228,000	10.50	2.80
41	Wiskott–Aldrich syndrome	2	15,000(6,000)	9.10(0.14)	4.6(5.90)	11,000	8.10	2.00
43	Immune thrombocytopenic purpura	1	170,000	9.60	3.00			
44	Immune thrombocytopenic purpura	2	356,000(139,000)	9.70(0.00)	2.9(1.13)	454,000	9.70	2.10
45	Immune thrombocytopenic purpura	3	199,000(21,000)	10.40(0.36)	2.77(0.45)	223,000	10.00	2.80
47	Immune thrombocytopenic purpura	1	274,000	11.30	4.50			
48	Immune thrombocytopenic purpura	1	130,000	11.90	5.80			
49	Immune thrombocytopenic purpura	7	388,000(19,000)	10.91(0.31)	2.7(0.51)	410,000	11.10	2.80
51	Immune thrombocytopenic purpura	3	227,000(140,000)	11.27(0.50)	5.57(2.71)	370,000	11.20	4.70
52	Immune thrombocytopenic purpura	1	37,000	12.70	8.90			
54	Immune thrombocytopenic purpura	7	84,000(49,000)	11.17(0.82)	5.24(1.49)	27,000	10.70	10.30
55	Wiskott–Aldrich syndrome	1	38,000	8.80	1.50			
56	Wiskott–Aldrich syndrome	1	57,000	9.00	4.60			
57	Immune thrombocytopenic purpura	1	17,000	10.90	5.20			
58	Immune thrombocytopenic purpura	3	32,000(23,000)	9.93(1.24)	7.90(7.59)	31,000	11.60	9.70
59	Immune thrombocytopenic purpura	1	125,000	11.00	1.90			
61	Immune thrombocytopenic purpura	3	24,000(3,000)	11.47(0.35)	6.83(2.76)	26,000	11.80	4.20
62	Wiskott–Aldrich syndrome	1	47,000	8.40	1.00			
63	Wiskott–Aldrich syndrome	3	25,000(6,000)	8.00(0.12)	3.18(1.59)	23,000	9.20	1.90
64	Wiskott–Aldrich syndrome	1	35,000	8.40	3.80			
65	Immune thrombocytopenic purpura	2	69,000(20,000)	11.42(0.86)	7.10(2.83)	55,000	12.90	10.40
67	Immune thrombocytopenic purpura	1	216,000	9.90	3.30			
68	Immune thrombocytopenic purpura	1	204,000	10.40	5.30			
69	Immune thrombocytopenic purpura	27	94,000(50,000)	11.60(0.84)	6.01(2.24)	46,000	10.50	4.90
70	Wiskott–Aldrich syndrome	1	34,000	8.20	1.70			
71	Wiskott–Aldrich syndrome	4	115,000(22,000)	8.83(0.05)	1.33(0.05)	94,000	8.80	1.40

*In the per-subject average data set (left-hand columns), each value is an average of all measures of the indicated parameter for each subject. For subjects with more than one blood draw, the SD of each average is given in parentheses. In the first-blood-draw data set (right-hand columns), the value of each parameter is from the first blood draw to report all three parameters for each subject*.

We used the first-blood-draw data set for the derivation cohort to compare the platelet parameters of the subjects with WAS to those of the subjects with a diagnosis of ITP. The IPF in subjects with WAS was significantly lower than the IPF in subjects with a diagnosis of ITP, although in both cases, the averages were within the range of normal. The average MPV was significantly lower in subjects with WAS than in subjects with a diagnosis of ITP, being below the normal range in the former case and within the normal range in the latter case. Platelet counts were also lower in the subjects with WAS than in the subjects with a diagnosis of ITP, although this difference was not statistically significant (Table 3).

**Table 3:** **Means (SDs) of platelet parameters from the first-blood-draw data set for the derivation cohort**.

	Platelets/mcL	MPV, fL	IPF, %
ITP	149,00 (121,000)	11.2 (1.4)	6.6 (5.2)
WAS	92,000 (69,000)	8.8 (0.6)	2 (1.2)
Range of normal	161,000–347,000	9.4–12.4	0.9–11.2
p	0.07	<0.0001	<0.0001

### Derivation of a rule to differentiate WAS and ITP

**Figure 1:**
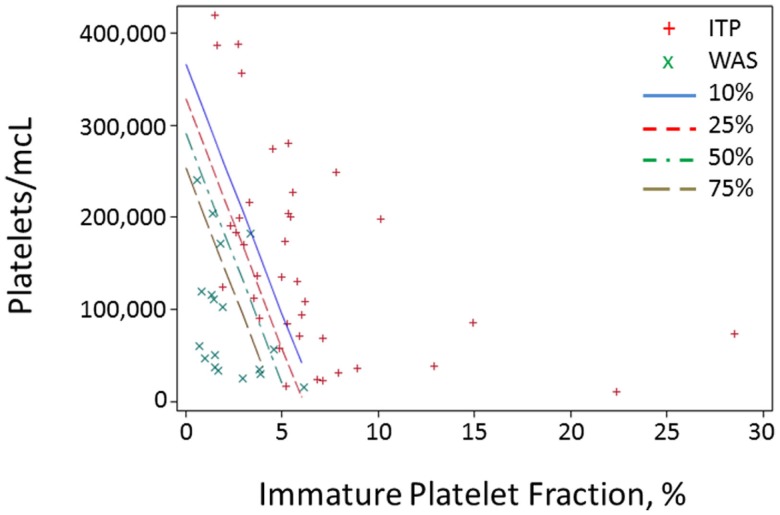
**Probability contours for a predictor based on IPF and platelet value**. For each subject in the derivation cohort, all blood draws with data on both platelet count and IPF were selected, and all values for the two parameters were averaged for each subject and plotted. Red crosses represent averages for ITP subjects and green *x*’s represent subjects with WAS. Contour lines represent different probabilities of a subject having WAS.

**Figure 2:**
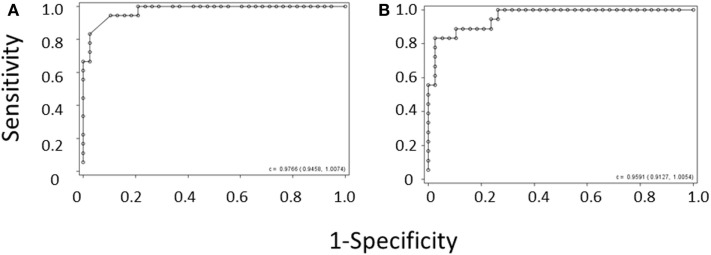
**ROC curve derived from (A) the MPV rule or (B) the IPF/PLT rule in the derivation cohort**. Each open circle marks the sensitivity and specificity of a given MPV cutoff. C-statistics appear in the lower right-hand corners of the graphs.

Because of the above-mentioned difficulties in obtaining MPV values during the diagnostic workup of thrombocytopenic patients, we attempted to develop a method of separating WAS and ITP based only on platelet count and IPF. We performed logistic regression of WAS/ITP status on IPF and platelet values in the per-subject average data set for the derivation cohort. The results of the logistic regression can be visualized by examining a scatter plot of average IPF vs. average platelet count in subjects with WAS or ITP (Figure 1). Contour lines based on the logistic regression indicate estimated probabilities of subjects to the left of the line having WAS. ROC curves were generated for the diagnosis of WAS based on MPV and for the diagnosis of WAS based on platelet count and IPF (Figures 2A,B). We arbitrarily chose a 10% probability of WAS as an appropriate level at which it would be reasonable to consider further testing to rule out WAS in a boy with isolated thrombocytopenia. Based on this decision, the logistic regression indicated that a tentative diagnosis of WAS could be made, to be followed by additional evaluation, when:

$$\left[\left(75\times \text{IPF}\right)+\text{Plt}\right]<500$$

where IPF is the IPF as a percentage and Plt is the platelet count expressed as thousands of platelets per microliter.

### Testing of the IPF/PLT rule and the MPV rule with a validation cohort

To obtain unbiased estimates of sensitivity and specificity of the IPF/PLT rule, we tested it against a subsequent independent data set of platelet parameters for subjects with WAS and ITP. The BTRIS system was again queried. All CBCs and IPF measurements from subjects with a discharge diagnosis of either ITP or WAS that were drawn between April 2, 2013 and March 19, 2014 were collected. This entire data set is presented in Table S3 in Supplementary Material. Blood draws with complete data (platelet count, MPV, and IPF) were selected, and are presented in Table S4 in Supplementary Material. This data set included 28 blood draws from 7 subjects with a diagnosis of ITP and 33 blood draws from 22 subjects with WAS. We used this data to create a per-subject average data set and a first-blood-draw data set for the validation cohort. These data sets are presented in Table 4.

**Table 4:** **Final data sets for the validation cohort**.

Subject	Discharge diagnosis	Number of blood draws	Per-subject average data set			First-blood-draw data set		
			Average platelets/mcL	Average MPV, fL	Average IPF, %	Platelets/mcL	MPV, fL	IPF, %
2	Wiskott–Aldrich syndrome	1	266,000	9.50	1.80	266,000	9.5	1.8
3	Wiskott–Aldrich syndrome	1	175,000	9.30	2.40	175,000	9.3	2.4
4	Wiskott–Aldrich syndrome	1	191,000	10.50	2.70	191,000	10.5	2.7
5	Wiskott–Aldrich syndrome	2	57,000(16,000)	8.1(0.14)	1.3(0.85)	46,000	8.2	1.9
6	Wiskott–Aldrich syndrome	1	259,000	10.50	3.00	259,000	10.5	3
7	Immune thrombocytopenic purpura	1	124,000	10.00	5.50	124,000	10	5.5
9	Immune thrombocytopenic purpura	3	187,000(17,000)	11.73(0.06)	9.3(0.26)	207,000	11.7	9.2
11	Wiskott–Aldrich syndrome	1	192,000	10.10	2.60	192,000	10.1	2.6
12	Wiskott–Aldrich syndrome	1	89,000	8.90	1.40	89,000	8.9	1.4
13	Wiskott–Aldrich syndrome	3	122,000(16,000)	9.47(0.23)	1.6(0.44)	130,000	9.6	2.1
14	Wiskott–Aldrich syndrome	1	134,000	8.60	1.10	134,000	8.6	1.1
15	Wiskott–Aldrich syndrome	1	145,000	9.10	1.10	145,000	9.1	1.1
16	Wiskott–Aldrich syndrome	1	94,000	9.70	2.60	94,000	9.7	2.6
17	Immune thrombocytopenic purpura	1	423,000	9.90	1.70	423,000	9.9	1.7
18	Wiskott–Aldrich syndrome	1	224,000	10.20	2.50	224,000	10.2	2.5
19	Wiskott–Aldrich syndrome	1	20,000	7.80	2.30	20,000	7.8	2.3
20	Wiskott–Aldrich syndrome	1	214,000	10.10	2.50	214,000	10.1	2.5
21	Immune thrombocytopenic purpura	2	152,000(50,000)	10.65(0.07)	5.90(2.26)	187,000	10.7	4.3
23	Wiskott–Aldrich syndrome	4	30,000(10,000)	8.55(0.59)	2.23(0.61)	22,000	8	2
25	Immune thrombocytopenic purpura	1	152,000	10.10	2.60	152,000	10.1	2.6
26	Wiskott–Aldrich syndrome	1	33,000	8.80	4.40	33,000	8.8	4.4
27	Wiskott–Aldrich syndrome	2	155,000(15,000)	8.70(0.28)	0.95(0.07)	166,000	8.5	1
28	Wiskott–Aldrich syndrome	3	21,000(5,000)	9.57(0.90)	5.37(1.10)	25,000	9	5.3
30	Wiskott–Aldrich syndrome	1	40,000	8.10	3.00	40,000	8.1	3
31	Wiskott–Aldrich syndrome	2	40,000(5,000)	8.75(0.35)	1.30(0.00)	37,000	9	1.3
32	Wiskott–Aldrich syndrome	2	41,000(2,000)	8.6(1.00)	1.00(0.14)	39,000	8.9	1.1
33	Immune thrombocytopenic purpura	4	84,000(10,000)	12.18(0.73)	8.9(0.90)	70,000	11.1	7.9
34	Immune thrombocytopenic purpura	16	87,000(19,000)	10.21(0.46)	3.79(0.66)	81,000	10	4.1
35	Wiskott–Aldrich syndrome	1	104,000	9.20	0.70	104,000	9.2	0.7

*In the per-subject average data set (left-hand columns), each value is an average of all measures of the indicated parameter for each subject. For subjects with more than one blood draw, the SD of each average is given in parentheses. In the first-blood-draw data set (right-hand columns), the value of each parameter is from the first blood to report all three parameters for each subject*.

The IPT/PLT rule and the MPV rule were each evaluated on both the per-subject average data set and the first-blood-draw data set for the validation cohort by comparing the predicted diagnoses to the recorded discharge diagnoses. These results are presented in Table 5. Using the per-subject average data set, both rules significantly separated subjects with WAS from subjects with a diagnosis of ITP, with *p* values of 0.0084 for the MPV rule and 0.0002 for the IPF/PLT rule. Using the first-blood-draw data set, results were nearly identical, with *p* values of 0.0063 for the MPV rule and 0.0002 for the IPF/PLT rule.

**Table 5:** **Discharge diagnoses and diagnoses determined by the MPV rule or by the IPF/PLT rule for the validation cohort**.

Subject	Discharge diagnosis	Per-subject average data set		First-blood-draw data set	
		Diagnosis by MPV rule	Diagnosis by IPF/Plt rule	Diagnosis by MPV rule	Diagnosis by IPF/Plt rule
2	Wiskott–Aldrich syndrome	**ITP**	WAS	**ITP**	WAS
3	Wiskott–Aldrich syndrome	WAS	WAS	WAS	WAS
4	Wiskott–Aldrich syndrome	**ITP**	WAS	**ITP**	WAS
5	Wiskott–Aldrich syndrome	WAS	WAS	WAS	WAS
6	Wiskott–Aldrich syndrome	**ITP**	WAS	**ITP**	WAS
7	Immune thrombocytopenic purpura	ITP	ITP	ITP	ITP
9	Immune thrombocytopenic purpura	ITP	ITP	ITP	ITP
11	Wiskott–Aldrich syndrome	**ITP**	WAS	**ITP**	WAS
12	Wiskott–Aldrich syndrome	WAS	WAS	WAS	WAS
13	Wiskott–Aldrich syndrome	**ITP**	WAS	**ITP**	WAS
14	Wiskott–Aldrich syndrome	WAS	WAS	WAS	WAS
15	Wiskott–Aldrich syndrome	WAS	WAS	WAS	WAS
16	Wiskott–Aldrich syndrome	**ITP**	WAS	**ITP**	WAS
17	Immune thrombocytopenic purpura	ITP	ITP	ITP	ITP
18	Wiskott–Aldrich syndrome	**ITP**	WAS	**ITP**	WAS
19	Wiskott–Aldrich syndrome	WAS	WAS	WAS	WAS
20	Wiskott–Aldrich syndrome	**ITP**	WAS	**ITP**	WAS
21	Immune thrombocytopenic purpura	ITP	ITP	ITP	ITP
23	Wiskott–Aldrich syndrome	WAS	WAS	WAS	WAS
25	Immune thrombocytopenic purpura	ITP	**WAS**	ITP	**WAS**
26	Wiskott–Aldrich syndrome	WAS	WAS	WAS	WAS
27	Wiskott–Aldrich syndrome	WAS	WAS	WAS	WAS
28	Wiskott–Aldrich syndrome	**ITP**	WAS	WAS	WAS
30	Wiskott–Aldrich syndrome	WAS	WAS	WAS	WAS
31	Wiskott–Aldrich syndrome	WAS	WAS	WAS	WAS
32	Wiskott–Aldrich syndrome	WAS	WAS	WAS	WAS
33	Immune thrombocytopenic purpura	ITP	ITP	ITP	ITP
34	Immune thrombocytopenic purpura	ITP	**WAS**	ITP	**WAS**
35	Wiskott–Aldrich syndrome	WAS	WAS	WAS	WAS

*The left-hand columns use the per-subject average data set, and the right-hand columns use the first-blood-draw data set*.

*Assigned diagnoses in boldface differ from the discharge diagnoses*.

Sensitivities and specificities were calculated for each rule for each data set. Using either data set, the MPV rule was perfectly specific, with sensitivities of 0.59 for the per-subject average data set and 0.64 for the first-blood-draw data set. Using the IPF/PLT rule on either data set, sensitivity was 1 and specificity was 0.71.

**Figure 3:**
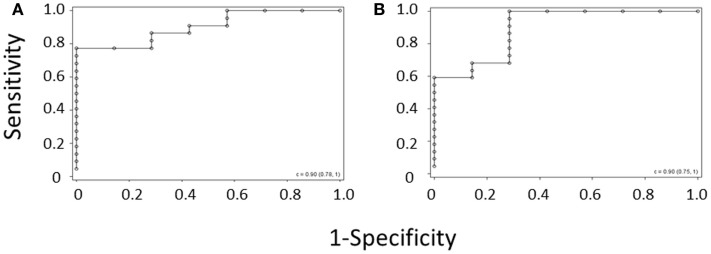
**ROC curve derived from (A) the MPV rule or (B) the IPF/PLT rule in the validation cohort**. Each open circle marks the sensitivity and specificity of a given MPV cutoff. C-statistics appear in the lower right-hand corners of the graphs.

ROC curves were again generated for the diagnosis of WAS based on MPV and for the diagnosis of WAS based on platelet count and IPF, using the per-subject average data set for the validation cohort (Figures 3A,B). C-statistics for the two curves were almost identical, showing that the IPF/PLT rule has overall diagnostic accuracy similar to the MPV rule.

## Discussion

WAS can often be separated from ITP on the basis of additional findings, including eczema and frequent infections (24). Diagnosis of a boy with newly recognized thrombocytopenia should take into account findings from history, physical examination, and laboratory evaluation. Nevertheless, the range of presentations of WAS is quite broad. Only a minority of patients present with the full Wiskott–Aldrich syndrome (27), whereas thrombocytopenia is almost always present (28). This makes the differential diagnosis of WAS and ITP clinically relevant. Because the use of MPV to distinguish WAS from ITP has a number of shortcomings, alternative strategies would be helpful. Our data show that a rule constructed using the platelet count and IPF can have greater sensitivity for the diagnosis of WAS than the traditional rule using only the MPV, while maintaining overall diagnostic accuracy.

Our data suggest that adding consideration of the IPF and platelet count in the diagnostic algorithm for thrombocytopenia in boys may allow for the detection of more patients with WAS than would be detected using MPV. In order to justify an increase in sensitivity at the expense of specificity, it is necessary to consider the clinical utility of ruling in or ruling out WAS in a boy newly found to have thrombocytopenia.

While the initial management of WAS with acute thrombocytopenic bleeding is similar to the initial management of ITP with acute thrombocytopenic bleeding, consisting of observation, IVIG, steroids, or some combination of these treatments, the long-term management and prognoses of the two diseases are quite different. For this reason, it might be appropriate to favor a diagnostic strategy that sacrifices specificity for sensitivity. Using the IPF/PLT rule for a theoretical population of 2,000,000 boys with thrombocytopenia (8 of whom would have WAS and 80 of whom would have ITP), no WAS patients would be missed, but 23 patients ITP would be initially diagnosed with WAS. Using the traditional rule on the same cohort, no patients with ITP would be felt to have WAS, but three diagnoses of WAS would be missed. The clinical decision then becomes whether it is preferable to initially misdiagnose eight boys with ITP as having WAS or to misdiagnose one boy with WAS as having ITP.

The above data show that subjects with Wiskott–Aldrich syndrome have a less robust thrombopoietic response to low platelet counts than do subjects with a diagnosis of ITP. In addition to the clinical utility of this observation, the data support the belief that WAS is a thrombocytopenia of both increased thrombocytolysis and decreased thrombopoiesis.

This study has a number of limitations. Foremost among these is the retrospective nature of the study. These data were not collected in order to evaluate the utility of using the IPF in addition to the platelet count and MPV in the differential diagnosis of WAS and ITP, but rather were obtained in the course of routine or investigational clinical care. Another limitation is that data were collected from a clinical database, rather than from a thorough review of the subject charts. While this approach fulfills the ethical imperative to maximize research subject privacy, the use of isolated laboratory data removed from the clinical context is expected to lead to some loss of data integrity. For example, the clinical circumstances in which these values were obtained, including the indication for blood draw, and perianalytical management with platelet transfusion or other measures, were not determined. It is possible, and in fact likely, that some of these blood draws were obtained after platelet transfusion, and thus the numbers observed may reflect the contribution of donor platelets to total platelet count, MPV, and IPF. If this is the case, however, one would expect such errors to be distributed similarly among subjects with both ITP and WAS. The effect of this would be to blunt the observed differences, and so the persistence of statistically significant differences between WAS and ITP subjects argues that this source of error did not lead to important confounding.

As for any study of a rare disease, the small number of subjects in the derivation cohort, and particularly in the validation cohort, is an inherent weakness of this study. Furthermore, the proportion of subjects with WAS in the two cohorts is not the same, likely reflecting changes in research interests at the hospital from which these data were derived. Gathering additional data in order to expand the validation cohort might offset these differences, but is not possible at this time.

A similar limitation in the data is the use of a unique population enriched in subjects with WAS. Both the WAS subjects and the ITP subjects seen at the single research facility from which data were obtained may not well represent the general population of patients with thrombocytopenia. This limitation is offset by having sufficient numbers of subjects with rare diseases to collect enough data for meaningful analysis in a timely fashion.

The NIH Clinical Center is a referral-based clinical research institution. As such, it is common that patients are seen after initial diagnosis and treatment of the underlying disease. This can explain why for some of the subjects analyzed in this study, the platelet counts are higher than what one would expect for patients newly presenting with ITP or WAS. Indeed, it is likely that these patients were seen at the NIH Clinical Center after their initial diagnosis of thrombocytopenia and, possibly, also after treatment of low platelets had been initiated. Nevertheless, we observed a significant difference in IPF between subjects with ITP and subjects with WAS, which is a strength of the study and indicates that the proposed rule is robust.

With respect to diagnostic attribution, only subjects with discharge diagnoses of WAS or ITP were considered. Other potential subjects could have been identified by accepting admission diagnoses or entries in problem lists as qualifying diagnoses. This choice was not made because it was felt that discharge diagnoses would be better substantiated than these other sources of diagnosis. Because ITP is a diagnosis of exclusion, it is also possible that some of the subjects identified as having ITP in their discharge diagnoses did not, in fact, have the disease, but had another cause of thrombocytopenia. Additional efforts to substantiate the diagnosis of ITP would require individual review of medical records, which we sought to avoid as noted above.

Another limitation is the handling of the different number of blood-draws for each subject. We used two different strategies to address this, either looking at the average value of each parameter for each subject or looking at the first-blood-draw with complete data for each subject. Results of our analyses were similar using either strategy. An alternative might have been to use a generalized linear mixed model in place of logistic regression, although this is a more complex analysis and requires additional assumptions.

Not all hematology analyzers routinely report the IPF. This may limit the generalizability of our study to some extent until instrumentation updates are widespread.

Because patients with WAS can also genuinely develop ITP, another concern would be the applicability of our rule to the diagnosis of a patient with WAS and ITP. One might expect that ITP in a WAS patient would not resolve the platelet production defect of WAS. But, a sudden large decrease in platelets might allow for a WAS patient to mount a thrombopoietic response with a normal, rather than low, IPF. The moderate efficacy of eltrombopag in WAS suggests that the megakaryocytes of WAS patients respond somewhat to thrombopoietin receptor stimulation, although this response may not be entirely normal. We were unable to evaluate this clinical situation without chart review, and the actual thrombopoietic response of patients with WAS to acute ITP remains an important research question.

The present study has a number of strengths as well. The use of electronically collected and archived data, as opposed to manual chart review, may have increased the likelihood that all relevant subjects were captured in the initial cohort. Refraining from chart review also has the benefit of preventing arbitrary exclusion of subjects based on data other than platelet count, MPV, IPF, and discharge diagnosis. The fact that these data were collected retrospectively from actual clinical records, rather than prospectively as planned research, argues for the applicability of the derived rules to a “real-life” clinical setting. A second strength is the validation of the proposed rule in a subsequent and independent validation cohort.

The use of IPF in the differential diagnosis of WAS and ITP is appealing for several practical reasons. The IPF can be reported regardless of the platelet count, which is not the case for the MPV. The parameter is typically available any time a CBC is run on a hematology analyzer designed to capture it. Therefore, no extra blood is required to run the test. As a numerical rather than a qualitative evaluation, the use of IPF does not require expertise in the evaluation of peripheral blood smears and thus may allow a non-hematologist to suspect the diagnosis of WAS. There is also intuitive appeal to using IPF in a manner analogous to the role of the reticulocyte count in the evaluation of anemia. An even simpler rule of thumb would be to consider WAS only in thrombocytopenic patients whose IPF is not elevated. No WAS subject in our study had a frankly elevated IPF, whereas 4 of 38 subjects with a diagnosis of ITP had this finding. While this rule is perfectly sensitive, the specificity is barely more than 10%.

There are additional considerations in applying our data to the clinical care of boys with newly diagnosed thrombocytopenia. The reticulated platelet percentage (an analyte similar to the IPF) is not routinely used to diagnose ITP, and in a recent consensus document, the reticulated platelet count was said to be of unproven or uncertain benefit in the diagnosis of ITP (29). Our data do not support using the IPF to confirm a diagnosis of ITP, but rather using the IPF and platelet count to rule out the diagnosis of WAS with enough confidence to avoid further testing. Should a child be found to have a low IPF in the setting of newly recognized thrombocytopenia, referral to an immunologist should be considered for the purpose of clinical correlation with signs or symptoms of immune dysregulation, such as eczema, infections, atopy, and either a personal or family history of immunodeficiency.

While it is reasonable to assume that in a patient without a pathologic cause of thrombocytopenia, low normal platelets would be associated with an IPF in the upper part of the normal range, we cannot rule out that situations may exist in which healthy subjects with no known platelet disorder could present low normal platelet counts and low normal IPF, such that applying the IPF/PLT rule would result in values consistent with the diagnosis of Wiskott–Aldrich syndrome. Therefore, it seems advisable to limit the application of the IPF/PLT rule to patients who have thrombocytopenia, or who have a history of low platelet counts.

While this study focuses on the clinically important differential diagnosis of ITP and WAS, the use of IPF may allow for separation of other inherited or acquired thrombocytopenias from ITP. There is no *à priori* reason to suspect that this would not be possible, but data analogous to these data will need to be collected to formally evaluate the use of IPF, or the IPF/PLT rule, in the differential diagnosis of other inherited or acquired thrombocytopenias from ITP. Such a study has been done suggesting that the absolute IPF value could be used to distinguish ITP from acute lymphocytic leukemia (ALL) and the IPF percentage to distinguish ITP from hypoproliferative thrombocytopenias including Fanconi anemia, thrombocytopenia with absent radii, severe aplastic anemia, and myelodysplastic syndrome (30).

In summary, the IPF is a convenient and readily available platelet parameter. Its use can help to increase sensitivity of the diagnosis of Wiskott–Aldrich syndrome, and potentially other thrombocytopenias due to decreased production. This in turn may spare children with these somewhat less common causes of thrombocytopenia from ill-advised attempts at immunosuppression while allowing them to access appropriate, and potentially definitive or life-saving, therapy in a timely manner. The data presented in this article are derived from an admittedly atypical but nevertheless clinical cohort of subjects with thrombocytopenia. If additional studies, including studies using prospectively collected data, are consistent with the current study, the IPF/PLT rule or another similar derivation can be validated as an appropriate first-line study in the diagnosis of newly recognized thrombocytopenia.

## Author Contributions

RS made the initial clinical observations, developed the idea for the study, collected and analyzed data, and wrote the first draft of the manuscript. NO performed statistical analysis, derived the prediction rule by logistic regression, and constructed ROC curves. FC supervised the research and edited the manuscript. RS and FC provided clinical care for the patients with WAS. All authors reviewed the manuscript for intellectual content.

## Conflict of Interest Statement

Emmes Corporation is a contract research organization that received payment for Neal Oden’s participation in this work. Robert Sokolic and Fabio Candotti have no conflict of interest to declare.

## Supplementary Material

The Supplementary Material for this article can be found online at http://journal.frontiersin.org/article/10.3389/fped.2015.00049/abstract

Table S1 **Complete data for the derivation cohort**. NR, not reported; ND, not done.

Table S2 **Blood draws with complete data for the derivation cohort**.

Table S3 **Complete data for the validation cohort**. NR, not reported; ND, not done.

Table S4 **Blood draws with complete data for the validation cohort**.
